# Identification of Novel Rodent-Borne Orthohantaviruses in an Endemic Area of Chronic Kidney Disease of Unknown Etiology (CKDu) in Sri Lanka

**DOI:** 10.3390/v13101984

**Published:** 2021-10-02

**Authors:** Devinda S. Muthusinghe, Kenta Shimizu, Sithumini M. W. Lokupathirage, Zhouoxing Wei, Yomani D. Sarathkumara, G. R. Amanda Fonseka, Pavani Senarathne, Nobuo Koizumi, Tomonori Kawakami, Akio Koizumi, Chaminda Wickramasinghe, Hideki Ebihara, Keita Matsuno, Yoshimi Tsuda, Jiro Arikawa, Chandika D. Gamage, Kumiko Yoshimatsu

**Affiliations:** 1 Graduate School of Infectious Diseases, Hokkaido University, Sapporo 060-0818, Japan; devindasm@med.hokudai.ac.jp (D.S.M.); sithuminilokupathirage@czc.hokudai.ac.jp (S.M.W.L.); lamtuanglavaron@gmail.com (Z.W.); 2 Department of Microbiology and Immunology, Faculty of Medicine, Hokkaido University, Sapporo 060-8638, Japan; kshimizu@med.hokudai.ac.jp (K.S.); tsuday@nagasaki-u.ac.jp (Y.T.); arikawaj@nagasaki-u.ac.jp (J.A.); 3 Department of Microbiology, Faculty of Medicine, University of Peradeniya, Peradeniya 20400, Sri Lanka; yomani.sarathkumara@my.jcu.edu.au (Y.D.S.); gramandafonseka@gmail.com (G.R.A.F.); pavanisenarathne@gmail.com (P.S.); 4 Australian Institute of Tropical Health and Medicine, James Cook University, Cairns, QLD 4878, Australia; 5 Department of Bacteriology I, National Institute of Infectious Diseases, Tokyo 162-8640, Japan; nkoizumi@niid.go.jp; 6 Department of Environmental and Civil Engineering, Faculty of Engineering, Toyama Prefectural University, Toyama 939-0398, Japan; kawakami@pu-toyama.ac.jp; 7 Department of Health and Environmental Sciences, Kyoto University Graduate School of Medicine, Kyoto 606-8501, Japan; koizumi@kyoto-hokenkai.or.jp; 8 Postgraduate Institute of Science, University of Peradeniya, Peradeniya 20400, Sri Lanka; wmcwick@gmail.com; 9 Department of Molecular Medicine, Mayo Clinic, Rochester, MN 55905, USA; hebihara@niid.go.jp; 10 International Institute for Zoonosis Control, Hokkaido University, Sapporo 001-0020, Japan; matsuk@czc.hokudai.ac.jp; 11 Institute for Genetic Medicine, Hokkaido University, Kita-ku, Kita-15, Nishi-7, Sapporo 060-0815, Japan

**Keywords:** hantavirus, *Mus booduga*, *Thailand orthohantavirus*, Anjozorobe hantavirus

## Abstract

We reported the genetic evidence of circulating hantaviruses from small mammals captured in a chronic kidney disease of unknown etiology (CKDu) hotspot area of Sri Lanka. The high seroprevalence of anti-hantavirus antibodies against *Thailand orthohantavirus* (THAIV) has been reported among CKDu patients and rodents in Sri Lankan CKDu hotspots. We captured 116 small mammals from CKDu endemic regions in the Polonnaruwa District of Sri Lanka. Seven animals (five out of 11 *Mus booduga* and two out of 99 *Rattus rattus*) were PCR-positive for the hantavirus. A rat-borne sequence was grouped with a THAIV-like Anjozorobe virus. In contrast, *Mus*-borne sequences belonged to the THAIV lineage, suggesting a novel orthohantavirus species according to the phylogenetic analyses and whole-genome comparisons. Our genetic evidence indicates the presence of two THAIV-related viruses circulating in this CKDu endemic area, suggesting a basis for further investigations to identify the infectious virus in patients with CKDu and the CKDu induction mechanism of these viruses.

## 1. Introduction

A previously unexplained form of renal disease, referred to as a chronic kidney disease of unknown etiology (CKDu), has been increasingly diagnosed over the past three decades in dry zone areas of Sri Lanka, becoming an overwhelming public health burden [1]. This disease has become more prevalent among rural agricultural communities [2], where males are more often affected than females [3]. Affected individuals show no symptoms until the disease progresses into its late stages. Areas in 13 out of 25 districts in the country have been identified as high-risk regions for the occurrence of CKDu. North Central Province alone has reported approximately 20,000 CKDu patients with a population prevalence rate of 4.7% [4]. The scarcity of recent incidence data has made it difficult to understand the current prevalence of CKDu in the country. Moreover, despite many studies conducted over the past few decades, the etiology of CKDu remains obscure.

Hantaviruses are a group of zoonotic pathogens belonging to the family Hantaviridae of the order Bunyavirales. The spherical enveloped viral particles consist of a tri-segmented negative-strand RNA genome. The large (L), medium (M), and small (S) genome segments encode an L-protein, a glycoprotein precursor (GPC) of two envelope glycoproteins Gn and Gc, and a nucleocapsid protein (N), respectively [5]. Hantaviruses currently have a relatively diverse host range, with rodents, shrews, moles, and bats being the common hosts. Interestingly, all medically important human pathogenic hantaviruses are carried by rodent hosts [6]. Hemorrhagic fever with renal syndrome (HFRS) in Eurasia and hantavirus cardiopulmonary syndrome (HCPS) represents two severe forms of human infections caused by hantaviruses. HCPS shows a higher fatality rate (25–35%) than HFRS in Asia (5–15%) [7]. East Asia accounts for approximately 90% of HFRS cases caused by Old World orthohantaviruses, such as the Hantaan virus (HTNV) and Seoul virus (SEOV) [8]. Southeast Asia, South Asia, and the Indian oceanic region are home to the *Thailand orthohantavirus* (THAIV) [9] and its genetic variants (the Anjozorobe (ANJZV) [10], Serang [11], Jurong [12], and Mayotte [13] viruses). The pathogenicity of these viruses remains unexplained because of the lack of data. Although several sero-epidemiological reports have described human infections involving THAIV in Thailand, India, and Sri Lanka [14,15,16] and ANJZV in Madagascar [17], there are no confirmed clinical cases of HFRS or HCPS documented in South Asia or Southeast Asia. Epidemiological information on hantaviruses and their hosts is limited, particularly in South Asian countries [8].

Hantavirus infection was first documented in Sri Lanka as early as 1988 by Vitarana and colleagues [18]. Since then, very few reports have been published on individuals with suspected leptospirosis who have been found to possess anti-hantavirus antibodies [19,20]. It was recently reported by Gamage et al. that 72 (54.5%) out of 132 CKDu patients from the CKDu endemic area of Girandurukotte, Sri Lanka harbored antibodies against hantaviruses [21]. The existence of THAIV- or THAIV-related hantavirus infections was confirmed by serotyping 89 anti-hantavirus antibody-positive human serum samples obtained from the same area [22]. Similarly, high levels of antibodies against the hantavirus were reported among CKDu patients from a CKDu hotspot in Polonnaruwa District in the North Central Province of Sri Lanka [23]. In addition, a cross-sectional study carried out with case-control comparisons in two geographically distinct CKDu endemic areas vs. a nonendemic area in Sri Lanka demonstrated that exposure to the hantavirus was an independent risk factor associated with renal disease in the CKDu endemic regions [24]. An ecoepidemiological study in Girandurukotte serologically confirmed that THAIV-like hantavirus species were highly prevalent among the *Rattus rattus* lineage [25]. Serological findings from both humans and rodents in the CKDu areas supported the hypothesis that exposure to hantaviruses is a risk factor for the possible development of CKDu in Sri Lanka [26]. However, no studies have provided the genomic evidence from hantavirus rodent hosts circulating in Sri Lanka. Viral genomic information is essential in developing specific diagnostics to detect hantavirus infections in CKDu patients. The results will add further insights into the relationship between exposure to a hantavirus and CKDu etiology. Therefore, the current study aimed to address this knowledge gap. Hence, this report describes a genetic analysis of small mammals captured from a CKDu endemic area in Sri Lanka to determine the hantavirus species and possible natural hosts.

## 2. Materials and Methods

### 2.1. Sample Collection

**Figure 1:**
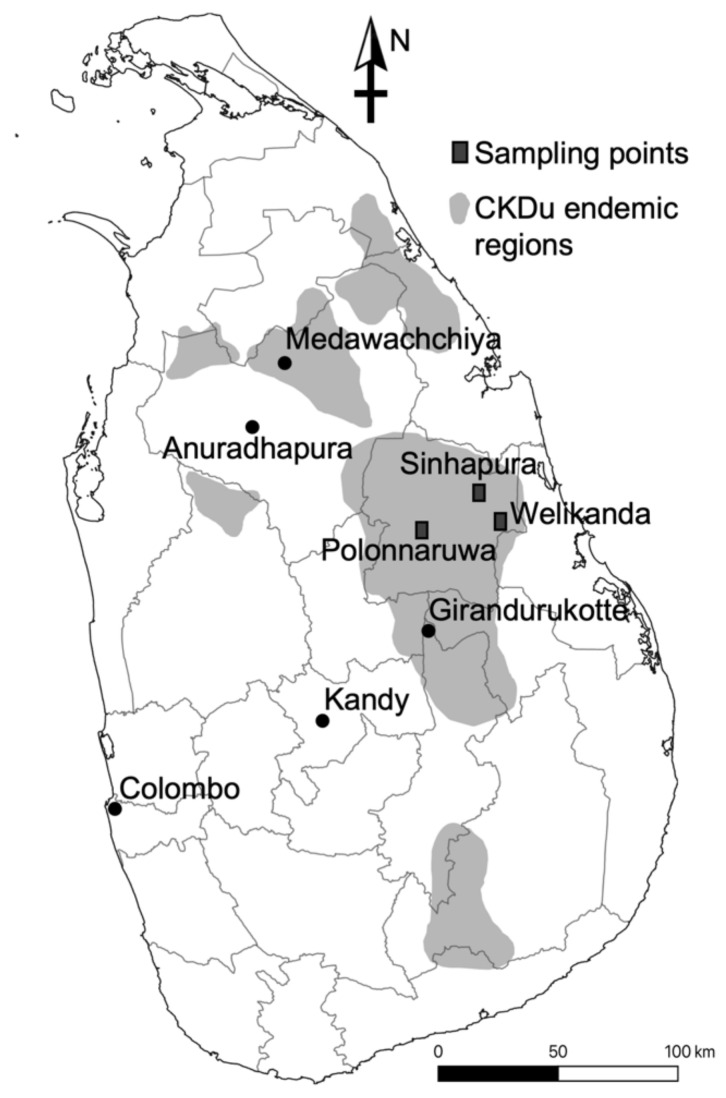
Map of Sri Lanka showing the CKDu endemic regions and sampling points of the study.

Small mammal samples were collected in September 2018 and July 2019 from the Polonnaruwa, Welikanda, and Sinhapura areas in Polonnaruwa District, where CKDu is highly prevalent (Figure 1). The study protocol was approved by the Ethics Committee of the Faculty of Veterinary Medicine and Animal Sciences of the University of Peradeniya, Sri Lanka (VER-16-007). In September 2018, rodent trapping was performed using cage-type traps to capture the first 98 rodents. Most of the traps used in July 2019 were Sherman traps (H. B. Sherman Traps, Inc., Tallahassee, FL, USA), and 18 additional rodents and shrews were collected. The captured species were initially identified based on their morphology. The animals’ body weight, sex, and other body parameters were recorded. The lungs, liver, kidneys, and blood samples from a heart puncture were collected from each animal. Parts of the lung and kidney tissues were preserved in RNAlater (Qiagen, Hilden, Germany), and a portion of the kidneys were preserved in 99.5% ethanol (Sigma-Aldrich, Burlington, MA, USA).

### 2.2. DNA Extraction and Rodent Species Identification

The DNA was extracted from small mammal kidney tissues preserved in ethanol using the DNAzol reagent (Invitrogen, Thermo Fisher Scientific, Carlsbad, CA, USA) according to the manufacturer’s instructions. PCR was performed on kidney DNA samples to amplify a mitochondrial cytochrome b (*cytb*) gene using AmpliTaq Gold^®^ 360 DNA polymerase (Applied Biosystems, Life Technologies, Warrington, UK) and the primers L14115, H15300, L497A, and H655A [27,28]. The PCR program consisted of 10 min of initial denaturation at 95 °C; 35 cycles of 95 °C for 30 s, 55 °C for 30 s, and 72 °C for 30 s; and a final extension at 72 °C for 7 min. The nucleotide sequences of the amplified *cytb* fragments were determined using a BigDye Terminator v3.1 cycle sequencing kit (Applied Biosystems) and a 3130xl Genetic Analyzer (Applied Biosystems).

### 2.3. Indirect Immunofluorescence Assay (IFA)

Anti-hantavirus IgG antibodies were detected in small mammal sera using IFAs based on antigens from THAIV-infected and recombinant THAIV N protein-expressing Vero E6 cells, as described elsewhere [29]. Alexa Fluor 488-conjugated goat anti-rat IgG (for rat and *Bandicota* sera), anti-mouse IgG (for mouse sera) (Invitrogen), and protein A (for shrew and gerbil sera) were used as the secondary antibodies. Each serum sample was diluted 1:100 in PBS. Scattered granular immunofluorescence patterns in the cell cytoplasm were considered to indicate positive staining.

### 2.4. RNA Extraction, cDNA Synthesis, and Hantavirus Screening PCR

RNA extraction was performed from lung and kidney tissues of all the small mammals preserved in RNAlater using the RNeasy Plus mini kit (Qiagen) following the manufacturer’s instructions. cDNA synthesis from the total RNA was carried out using the SuperScript IV VILO Master mix (Invitrogen). All lung cDNA samples were screened by PCR using AmpliTaq Gold^®^ 360 DNA polymerase and degenerate primers [30] targeting a conserved domain of the L genome segment of hantaviruses. The HAN-L-F2 (5′-TGCWGATGCHACIAARTGGTC-3′) and HAN-L-R1 (5′-AACCADTCWGTYCCRTCATC-3′) primers were used for the first round, followed by hemi-nested amplification using the HAN-L-F2 and HAN-L-R2 (5′-GCRTCRTCWGARTGRTGDGCAA-3′) primers. Both amplification reactions included 10 min of initial denaturation at 95 °C; 35 cycles of 95 °C for 30 s, 55 °C for 30 s, and 72 °C for 30 s; and a final extension at 72 °C for 7 min. Amplified PCR products with correct sizes were purified and sequenced as described previously.

### 2.5. Genomic Sequencing

All the screening PCR-positive samples were selected for hantavirus whole-genome sequencing via either the primer walking method or Illumina MiSeq sequencing. In the primer walking method, the primers were designed for all three genomic segments based on the initial sequences obtained in this study and previously published Muridae-borne hantavirus sequences (Supplemental Tables S1–S3) and were used to amplify segments of the genome, not including the termini. The PCR products were gel-purified and sequenced by Sanger sequencing, as described above.

For the Illumina MiSeq analysis, the RNA fractions extracted from lung tissues, as described above, were treated with the Ribo-Zero rRNA removal kit (Illumina, San Diego, CA, USA) to deplete host-derived rRNA. The treated RNAs were employed to construct sequencing libraries using the KAPA RNA HyperPrep kit (for Illumina) and the KAPA Dual-Indexed adapter kit (KAPA Biosystems, Wilmington, MA, USA). Twenty-four libraries and other nonrelated samples were mixed in equal amounts to obtain 9 fmol of a MiSeq library, which was then sequenced on the Illumina MiSeq platform using the MiSeq reagent kit v3 (Illumina) with 2 × 300-bp paired-end read lengths.

Since there is no reported complete sequence of the prototype THAIV L segment available for the whole-genome comparison, the entire L segment sequence of THAIV strain-749 (LC553715) was determined using the cDNA of the virus. The primer walking method was carried out using degenerate primers designed as described above (Supplemental Table S3), and the amplicons were sequenced by Sanger sequencing, as described previously. To complete the terminal sequences, the RACE method was applied as previously described [31] using the adapter sequences [31] and specific primers shown in Supplemental Table S3.

### 2.6. Sequence Alignment and Phylogenetic Analysis

The sequences obtained via Sanger sequencing were manually edited and aligned with reference genome sequences obtained from DNA databases. At the same time, the MiSeq reads were mapped onto reference genomes using GENETYX-MAC version 20.1.0 (Genetics Co., Ltd., Tokyo, Japan). The full-length sequences obtained from the S, M, and L segment ORFs aligned with representative sequences from other Muridae-borne hantaviruses using MUSCLE, as implemented in Geneious Prime^®^ 2020.2.2 (Biomatters, Ltd., Auckland, New Zealand). Multiple sequence alignments were edited and used to construct Bayesian phylogenetic trees using the MrBayes 3.2.6 [32] plug-in of Geneious Prime^®^ 2020.2.2 with the GTR + G + I substitutional model. Consensus cladograms were constructed using viral N protein amino acid sequences, and host *cytb* sequences were compared for the degree of concordance using Dendroscope V3.7.2. [33] to describe the coevolutionary relationships between the hantaviruses and hosts identified in this study, along with other representative rodent-, mole-, shrew-, and bat-borne hantaviruses and their hosts.

### 2.7. Quantification of Viral RNA

Whole-genome-positive rodent lung and kidney cDNAs were subjected to a quantitative real-time PCR analysis. For the *Mus* cDNA samples, primers LANS_F (5′-GAGAGCATGCCAGGGGTGCAGG-3′) and LANS_R (5′-GTAGGTGGACACCTATCAGGAGC-3′) were used. For the *R. rattus* cDNA samples, primers SA108S_F (5′-GATCATGCTAGGGATGCTGG-3′) and SA108S_R (5′-GTAGGAGGACACCGATCAGGTGC-3′) were used, with the KAPA SYBR FAST qPCR master mix (KAPA Biosystems) and a Light Cycler 480 instrument II (Roche, Indianapolis, IN, USA) according to the manufacturer’s instructions.

## 3. Results

### 3.1. Animal Species Identification

Morphological identification showed that the most (99/116) of the captured small mammals were *Rattus rattus*. An analysis of the *cytb* sequences from several animals confirmed that they belonged to lineage Ib, a Sri Lankan endemic lineage of *R. rattus* [25,28]. Eleven animals were identified as *Mus booduga* (Little Indian field mouse) after analyzing the *cytb* sequences (Supplemental Figure S1). We identified two clusters of *M. booduga* sequences in the phylogeny, which differed from the *M. booduga* sequences from India and Nepal. The other rodent and shrew species captured in this study were *Tatera indica* (Indian Gerbil) (*n* = 3), *Bandicota bengalensis* (*n* = 1), *Bandicota indica* (*n* = 1), and *Crocidura horsfieldii* (*n* = 1) (Table 1).

### 3.2. Sero-Survey and Hantavirus Screening PCR

As shown in Table 1, a total of 36.4% (40/116) of the captured animals were seropositive for anti-hantaviral antibodies in an IFA. Thirty-four out of 99 *R. rattus* individuals were seropositive, as were 5/11 *M. booduga* and 1/1 *B. bangelensis*. Genome screening was performed for all the small mammal lung cDNA samples. Out of 116 captured animals, seven were positive by the hantavirus genome screening PCR (Table 1). Positive amplicons were obtained from *M. booduga* (5/11) and *R. rattus* (2/99). All five seropositive *M. booduga* were PCR-positive, resulting in a high positive rate of 45.5% (5/11). Conversely, among 34 seropositive *R. rattus*, only one was PCR-positive. PCR positivity was also detected in a seronegative *R. rattus* individual. Whole-genome sequencing was carried out to determine the respective hantavirus species precisely.

### 3.3. Whole-Genome Sequencing

We determined nearly complete whole-genome sequences of six of the seven PCR screening-positive samples using the primer walking and MiSeq approaches. The accession numbers of the genome segment sequences determined in this study are listed in Supplemental Table S4. All *Mus*-borne hantavirus sequences were similar in the sequence identities and showed less similarity to those of all known THAIV-like viruses. Therefore, these *Mus*-borne sequences were designated as the Lanka virus. The sequences determined from *M. booduga* sample #98 (PR98) were used to represent Lanka viruses for further analyses, as it was the first *Mus* sample to obtain the whole genome of the Lanka virus. We failed to determine the whole-genome sequence from the seronegative rat (#32) that was positive according to PCR screening, and its amplicon sequence was identical to that of the Lanka virus. Sequence comparisons with other representative Muridae-born hantaviruses revealed that PR98 was the closest to ANJZV, and its S, M, and L segment open reading frames (ORFs) showed 62.6–80.1%, 59.4–76.9%, and 74–79.7% nucleotide identities, respectively, while the encoded N, GPC, and L proteins showed 61.4–93.2%, 53.6–87.2%, and 68.5–94.5% amino acid identities, respectively (Table 2). Another seropositive rat (#108) carried sequences differing from those of the Lanka virus. The sequence analysis of this *R. rattus-*borne virus, designated as strain SA108 (SA108; Sri Lankan ANJZV detected from rat #108) showed high similarity to ANJZV, a genetic variant of THAIV in the *R. rattus* species from the Madagascar Islands. SA108 led to a similar sequence identity range (Supplemental Tables S5–S7). The predicted GPC cleavage site, having a conserved WAASA motif, could be observed at amino acid positions 642–646 in both strains. The novel Lanka virus detected from *M. booduga* showed a high divergence from all the known THAIV-like viruses at both nucleotide and amino acid levels. The M segment nucleotide and amino acid sequences of the Lanka virus showed the lowest identity with the THAIV and THAIV-like viruses (Table 2 and Supplemental Table S6). In contrast, those of the L segment showed the highest identity values (Table 2 and Supplemental Table S7).

### 3.4. Sequence Alignment and Phylogenetic Analysis

**Figure 2:**
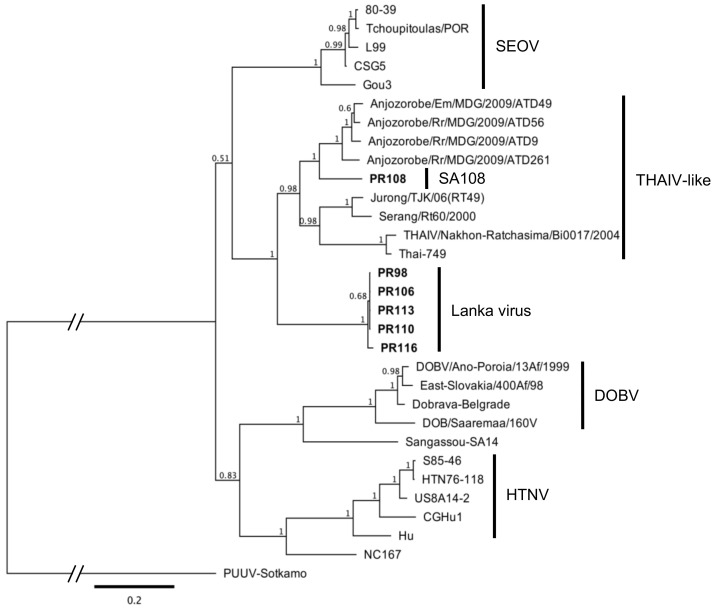
Phylogenetic tree based on S-segment ORF sequences representing the Muridae-borne hantaviruses and newfound viruses (shown in boldface) from this study. The scale bar indicates a sequence divergence of 0.2. The numbers above the nodes indicate the Bayesian posterior probability values. Hantaan (HTNV): S85-46 (AF288659), HTN76-118 (M14626), US8A14-2 (KU207208), CGHu1 (EU092218), and Hu (AB027111); Dabieshan: NC167 (AB027523); Seoul (SEOV): Gou3 (AF184988), L99 (AF288299), CSG5 (AB618112), Tchoupitoulas-POR (KU204960), and 80-39 (AY273791); Dobrava (DOBV): DOBV/Ano-Poroia/Afl9/1999 (AJ410615), Dobrava-Belgrade (L41916), East Slovakia/400Af/98 (AY168576), and DOB/Saaremaa/160V (AJ009773); Sangassou: SA14 (JQ082303); THAIV: Nakhon Ratchasima/Bi0017/2004 (AM397664), Thai-749 (AB186420), ANJZV strain Anjozorobe/Em/MDG/2009/ATD49 (KC490918), ANJZV strain Anjozorobe/Rr/MDG/2009/ATD56 (KC490916), ANJZV strain Anjozorobe/Rr/MDG/2009/ATD9 (KC490915), ANJZV strain Anjozorobe/Rr/MDG/2009/ATD261 (KC490914), Jurong strain TJK/06/RT49 (GQ274940), and Serang strain Serang/Rt60/2000 (AM998808); and Puumala (PUUV): Sotkamo (X61035).

**Figure 3:**
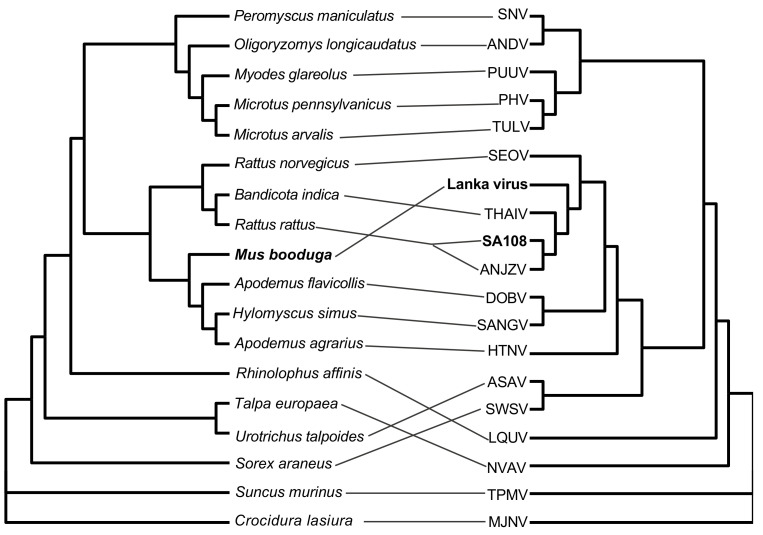
Tanglegram comparing the phylogenies of the representative hantaviruses and their hosts. The phylogeny of viruses based on amino acid sequences of the N protein (on the right) is compared with the *cytb* sequence-based phylogeny of their hosts (on the left). The newfound viruses are designated as the Lanka virus (LC553716) and SA108 (LC553722) in boldface, and the Lanka virus host *Mus booduga* (LC556235) is also shown in boldface. The other viruses used in the analysis include the shrew-borne thottimviruses Imjin virus (MJNV, KJ420559) from *Crocidura lasiura* (KJ004674) and Thottopalayam virus (TPMV, AY526097) from *Suncus murinus* (JF784171); the mole-borne *Nova mobatvirus* (NVAV, KR072621) from *Talpa europaea* (KF801566); the bat-borne *Longquan loanvirus* (LQUV, JX465422) from *Rhinolophus affinis* (DQ297582); the shrew-borne *Seewis orthohantavirus* (SWAV, KY651020) from *Sorex araneus* (AJ245893); the mole-borne *Asama orthohantavirus* (ASAV, EU929072) from *Urotrichus talpoides* (AB033611); the rodent-borne orthohantaviruses Seoul virus (SEOV, AY273791) from *Rattus norvegicus* (AB033713), the Thailand virus (THAIV, AM397664) from *Bandicota indica* (KJ592790), the Dobrava-Belgrade virus (DOBV, AJ410615) from *Apodemus flavicollis* (AY158445), the Sangassou virus (SANGV, JQ082300) from *Hylomyscus simus* (JX893846), the Hantaan virus (HTNV, M14626) from *Apodemus agrarius* (AB032851), the Sin Nombre virus (SNV, L25784) from *Peromyscus maniculatus* (JF489123), the Andes virus (ANDV, AF291702) from *Oligoryzomys longicaudatus* (KR822254), the Tula virus (TULV, Z49915) from *Microtus arvalis* (GU187363), the Prospect Hill virus (PHV, Z49098) from *Microtus pennsylvanicus* (KF948531), the Puumala virus (PUUV, X61035) from *Myodes glareolus* (FJ881480); and the THAIV genetic variant Anjozorobe virus (ANJZV, KC490918) from *Rattus rattus* (AB033702).

A phylogenetic analysis based on the ORFs of all three genomic segments of the SA108 and Lanka viruses clustered them with THAIV-like viruses (Figure 2 and Supplemental Figures S2 and S3). The Lanka virus showed a quite divergent topology in the phylogenetic trees, following the sequence identity results. The Lanka virus formed the basal clade in the S and M trees, where THAIV and its genetic variants seemed to diverge from the virus later. The tanglegram (Figure 3) illustrating the host–virus evolutionary relationships clearly showed the grouping of *M. booduga*, the Lanka virus host, with *Apodemus* and *Hylomyscus* species, which are the hosts of the Hantaan, Dobrava, and Sangassou orthohantaviruses found in Eurasia and Africa (Figure 3). The results revealed a notable difference since all the other THAIV-like hantavirus reservoir hosts were clustered into the *Bandicota* and *Rattus* groups (i.e., THAIV (*Bandicota indica*), ANJZV and Mayotte virus (*R. rattus*), Serang virus, and Jurong virus (*R. tanezumi*)).

### 3.5. Quantification of Viral RNA

**Figure 4:**
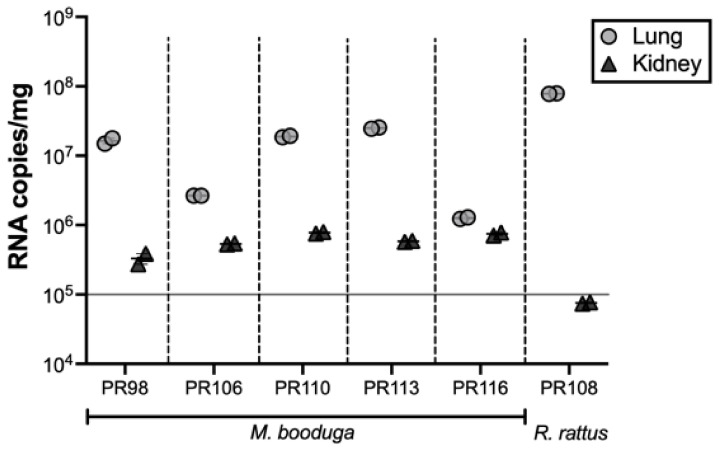
Viral RNA copy numbers in lung and kidney tissues of hantavirus genome-positive rodents. Tissues from *M. booduga* and *R. rattus* were examined by quantitative real-time PCR using the Lanka virus primer set and the SA108 primer set, respectively. The two markers of each sample show the two replicated runs of the same cDNA sample, and the error bars representing the standard error and the median of the duplicates are shown for each sample.

Higher viral RNA copy numbers were detected in lung tissues than in kidney tissues in all the rodent samples. *R. rattus* (PR108) had a notable difference in the viral RNA copy numbers between the two tissue types. All the *M. booduga* kidney tissues showed viral copy number values higher than 10^5^ copies/mg, while the single SA108-infected *R. rattus* kidney tissue sample showed a lower value (Figure 4).

## 4. Discussion

In this study, we report the detection of two novel hantaviruses, the Lanka virus and an ANJZV variant from Sri Lanka. The Lanka virus detected from *M. booduga* shows notable differences from all known THAIV genetic variants and from the THAIV prototype. The differences identified in the Lanka virus S and M segments and their corresponding proteins suggest the unique adaptation of this virus to its host, *M. booduga*. The S and M genomic sequences of the Lanka virus are placed as the basal branches of the THAIV-like clades in the corresponding phylogenetic trees, indicating that the Lanka virus might be the most ancient lineage of the THAIV-like hantaviruses. Based on the most recent proposed taxonomy guidelines, hantaviruses showing pairwise evolutionary distance (PED) values for the N protein and GPC concatenated amino acid sequences greater than 0.1 are considered distinct orthohantavirus species [34]. The corresponding values for the Lanka virus are 0.1344, 0.1214, and 0.1159 compared with the THAIV, ANJZV, and Jurong virus, respectively, suggesting that the Lanka virus is a novel, distinct orthohantavirus species. The tanglegram analysis further supported this hypothesis by accommodating the Lanka virus host in a different group of rodents from the usual THAIV-like virus hosts.

THAIV and its genetic variants, such as the ANJZV and Mayotte, Jurong, and Serang viruses, are carried primarily by *Bandicota* and *Rattus* species. Initially, rats were the targets of trapping, and we used only cage-type traps. As a result, many seropositive rats were captured, but a virus genome was not identified from any of them [25]. A partial Lanka virus genome was first detected from a seronegative rat (#32) after heminested PCR. This rat was thought to be in the early phase of infection. Seropositive but genome-negative rats were considered to have recovered from a spillover infection rather than representing a reservoir of the Lanka virus or SA108. Additionally, the fact that the rats can easily experience a spillover infection directly explains why humans living in the same field exhibit the same chance of infection as rats. A single mouse, #98, was captured in a cage-type trap, and this mouse seemed to be a hantavirus reservoir. After that, we switched to traps for capturing mice and ultimately succeeded in identifying the Lanka virus. All the genome-positive mice were captured within the Sinhapura area and had a relatively dispersed origin. *M. booduga* sample PR116 was collected from a location relatively far from the area where other genome-positive *M. booduga* samples were collected. The differences in the PR116-borne Lanka virus nucleotide sequences suggest the possible diversity among the Lanka viruses distributed in CKDu endemic areas and possibly in other regions as well.

To our knowledge, this is the first report providing genetic evidence of *Mus* species acting as hantavirus reservoir hosts. Several studies describing hantavirus genome detection in *Mus musculus* appear to represent spillover infections from reservoir hosts found in the same environmental habitats [35,36,37,38,39]. A perusal of the available literature revealed that the *M. booduga* species is distributed in East Pakistan, India, Southern Nepal, Sri Lanka, Bangladesh and Myanmar [40]. The finding that Sri Lankan *M. booduga* sequences are distinct from those found in India and Nepal (Supplemental Figure S1) indicates that the Sri Lankan *M. booduga* evolved as a distinct group, which segregated a long time ago from other strains in the Indian Peninsula. Strong coevolutionary relationships with natural hosts are often observed among the hantaviruses [41,42]. However, the identification of *M. booduga* as a host for a THAIV-like virus suggests that a host-switching event occurred long ago, resulting in the coevolution of the Lanka virus with the Sri Lankan *M. booduga* lineage. The detection of high viral RNA copy numbers in genome-positive rodent tissues suggests a high possibility of shedding viruses in their excreta. Yasuda et al. reported that the Seoul orthohantavirus was excreted in feces when showing more than 10^5^ genome copies/mg of lung tissues of *R. norvegicus* [43]. The Lanka virus and SA108 may pose elevated risks of human infections.

The two hantaviruses described herein were detected in CKDu endemic areas of Sri Lanka. It is of utmost importance to understand the epidemiological relationship between the virus infection and the prevailing human CKDu in the region. It is evident that young adult males engaged in farming activities year-round are significantly affected by CKDu in Sri Lanka. Male farmers working in agricultural fields are exposed to many external risk factors. We hypothesize that these individuals are exposed to hantaviruses in their working environments rather than in their homes. However, the infective virus and its source should be confirmed to determine possible interventions. Our study identified two candidate viruses and their distinct rodent hosts, which may transmit these viruses to humans in different habitats. *Rattus* species are well-adapted to peri-domestic environments and are thus distributed in both urban and rural areas. Their habitats are generally within houses or nearby neighborhoods, where they primarily feed on harvested crops stored inside homes or garbage dumps. On the other hand, *M. booduga*, commonly known as the little Indian field mouse, is most commonly found in agricultural fields, shrublands, and forest areas [39]. Therefore, the habitat of *M. booduga*, rather than that of *R. rattus*, is consistent with our hypothesized site of the acquiring a virus infection by humans. The geographic distribution of *M. booduga* includes India, where some regions are affected by CKDu. Hence, it is important to study whether Indian *M. booduga* strains can also carry hantaviruses that may confer a risk of CKDu.

CKDu has emerged as a significant public health problem in countries other than Sri Lanka, such as Nicaragua, El Salvador, and Costa Rica in Central America; some parts of India; and Egypt. Although the etiology has yet to be confirmed, extensive research has suggested some risk factors, such as heat/dehydration, infection/inflammation, and pesticides in Central American countries and water contamination/metals, pesticides, and infections in South Asian countries [44]. However, the hantavirus infection has, thus far, only been identified as a possible risk factor in CKDu patients from Sri Lanka [24,45,46].

Previous sero-epidemiological evidence indicated that THAIV-like hantaviruses infect both humans and rodents in CKDu hotspot regions. However, the unavailability of genetic information on hantaviruses circulating in the country has hindered the understanding of the relationship between these viruses and CKDu in Sri Lanka. Therefore, the current study aimed to fill the knowledge gap by identifying hantavirus genomes from rodent populations from a CKDu endemic region in Sri Lanka and further added a novel species to the list of hantavirus rodent hosts.

In conclusion, the current study revealed the genomic basis of hantaviruses in Sri Lanka. Our findings provided new insights for further investigations based on specific diagnostics for detecting the hantavirus species circulating among rodents and humans in other areas of Sri Lanka. These findings may contribute to better characterizing the exposure in CKDu patients to understanding the involvement of hantavirus infections in the context of pathophysiology of CKDu in Sri Lanka.

## Figures and Tables

**Table 1:** Summary of the captured species and test results.

Species	No. of Captured Animals	IFA Antibody (% Positive)	PCR (% Positive)
*Rattus rattus* complex	99	34 (34.3%)	2 (2%)
*Mus booduga*	11	5 (45.5%)	5 (45.5%)
*Tatera indica*	3	0	0
*Bandicota bengalensis*	1	1	0
*Bandicota indica*	1	0	0
*Crocidura horsfieldii*	1	0	0
Total	116	40	7

**Table 2:** Nucleotide and amino acid sequence identities of S, M, and L segment ORFs and their corresponding encoded proteins of the Lanka virus strains PR98 with SA108 and other representative Muridae-born hantaviruses.

	Lanka Virus Strain-PR 98					
	Nucleotide Identity, %			Amino Acid Identity, %		
	ORF_S	ORF_M	ORF_L	N	GPC	L-protein
THAIV	78.1	76.6	79.2	92.1	85.4	94.0
ANJZV	79.4	76.9	79.5	93.2	86.7	94.3
SA108	80.1	76.9	79.7	93.0	87.2	94.5
SEOV	75.0	72.3	76.7	85.8	79.7	88.3
HTNV	74.0	71.2	74.0	84.1	76.8	84.8
DOBV	72.6	70.7	74.2	83.2	75.7	85.6
PUUV	62.6	59.4	66.9	61.4	53.6	68.5

THAIV strain Thai-749 (S: AB186420, M: L08756, and L: LC553715); ANJZV strain Anjozorobe/Em/MDG/2009/ATD49 (S: KC490918, M: KC490919, and L: KC490922); Seoul virus (SEOV) strain 80-39 (S: AY273791, M: S47716, and L: X56492); Hantaan virus (HTNV) strain HTN76-118 (S: M14626, M: M14627, and L: X55901); Dobrava virus (DOBV) strain Dobrava-Belgrade (S: L41916, M: L33685, and L: JQ026206); and Puumala virus (PUUV) strain Sotkamo (S: X61035, M: X61034, and L: Z66548).

## Data Availability

Accession numbers of *cytb* sequences of hantavirus genome positive *Mus booduga* and *Rattus rattus* animals: LC556235 - LC556246, hantavirus genome sequences obtained from *Mus booduga* and *Rattus rattus* animals: LC553716 - LC553733, *Thailand orthohantavirus* strain Thai-749 L segment sequence: LC553715.

## Supplementary Materials

The following are available online at https://www.mdpi.com/article/10.3390/v13101984/s1: Figure S1: Phylogenetic tree based on *cytb* sequences of *Mus booduga* samples obtained in this study (in boldface) and other *Mus* spp. retrieved from databases. Figure S2: Phylogenetic tree based on M segment ORF sequences representing Muridae-borne hantaviruses and newfound viruses (in boldface) from this study. Figure S3: Phylogenetic tree based on L segment ORF sequences representing Muridae-borne hantaviruses and newfound viruses (in boldface) from this study. Table S1: List of primers used to amplify and sequence the S segment of the Lanka virus and SA108. Table S2: List of primers used to amplify and sequence the M segment of the Lanka virus and SA108. Table S3: List of primers used to amplify and sequence the L segment of the Lanka virus, SA108, and Thailand virus strain Thai-749. Table S4: Details of the accession numbers of *cytb* sequences and hantavirus genome sequences obtained from *Mus booduga* and *Rattus rattus* in this study. Table S5: Nucleotide and amino acid sequence identities of the S segment ORF and nucleocapsid protein of the novel viruses with representative Muridae-borne hantaviruses. Table S6: Nucleotide and amino acid sequence identities of the M segment ORF and glycoprotein precursor of the novel viruses with representative Muridae-borne hantaviruses. Table S7: Nucleotide and amino acid sequence identities of the L segment ORF and L protein of the novel viruses with representative Muridae-borne hantaviruses.
