# Supplementary material for: Identification of Novel Rodent-Borne Orthohantaviruses in an Endemic Area of Chronic Kidney Disease of Unknown Etiology (CKDu) in Sri Lanka
Source: Viruses. 2021 Oct 2;13(10):1984. doi: 10.3390/v13101984 (PMC8539126; doi:10.3390/v13101984)
Supplement: Supplementary file 1 [file viruses-13-01984-s001.zip › viruses-1386240-supplementary-1.pdf]

viruses

MDPI

Table S1. Lists of primers used for the amplification and sequencing of S segment of Lanka virus and SA108.

Specificity	Primer name	Sequence (5'-3')
Lanka virus specific primers	Lanka S607F	ATGAAAGCTGAGGAAATAACCCCAGG
	Lanka S620R	TCCTCAGCTTTCATGCTTGACTGGGC
	Lanka S682F	AGGAATATGGTCAGTCCAGTGATGAG
	Lanka S707R	CTCATCACTGGACTGACCATATTCCTAGC
	Lanka S1193F	TGTTTATGGTCTCCTGGGGAAAGGAAGCAG
	Lanka S1228F	AATTTTCACTTGGGAGATGACATGGACCC
Muridae-borne and THAIV-like primers	Muridae_hanta S1F	TAGTAGTAGICTICSTRAARAGCTAC
	Muridae_hanta S43F	AAAATGGCAACIATIGARGARITMC
	Muridae_hanta S370F	ATTGAIGARCCIACAGGMCARACIGC
	Muridae_hanta S974R	CCWGCAAAIACCCAIATYGAIGAIGG
	Muridae_hanta S1226R	TCIACWGCCTCYTTICCCCAIKCAACC
	Muridae_hanta S1322R	GGCTCITGRTTIGAIATYTCYTTIAC
	Muridae_hanta S1549R	AACTTAAYTAATTAACTTRATTAGTYAAC
	Muridae_hanta S1619R	ATATGGCAGTGATAATAATCAGTAG
	Muridae_hanta S1730R	GATCAAWTGAWATCTACATCCATATATAC
	Muridae_hanta S1887R	TAGTAGTAGTAKRCTCCCTAAARAGAC
	THAIV-like S416F	TCTAYYTGACATCITTTGTIATMCC
	THAIV-like S454F	GCACTGTAIATGYTIACRACRAGAGG
	THAIV-like S508F	ATGAGAATIMGITTYAAGGATGAYAG
	THAIV-like S562F	AAGCCAAARCAYCTITAYATTTCIATGCC
	THAIV-like S917R	GCATCYCTIGCATGRTCYCTTATICC
	THAIV-like S836R	TCTGCATTTGTIGGWGRRCTIGCATG

Table S2. Lists of primers used for the amplification and sequencing of M segment of Lanka virus and SA108.

Specificity	Primer name	Sequence (5'-3')
Lanka virus specific primers	Lanka M1722R	TGTGCTTTGAGCTCTTTCAAGGTCTC
	Lanka M1761R	AAGCAGTACGGACACTCAGATTGTGG
	Lanka M2090R	TGGAGCTTGAAGGAAGAGAGAAATC
	Lanka M2189F	CAGAACTTAGGGCACTGGTTTGATGC
	Lanka M2223R	TTAAGACGAGCATCAAACCAGTGCCC
	Lanka M2228F	AAAACCTCGTTCCATTGTTATGGGGC
	Lanka M2276R	AAGGATACTGGTACTTTGAACAGGC
Muridae-borne and THAIV-like primers	Muridae_hanta M1F	TAGTAGTAGACICCGCAARARAIAGCAG
	Muridae_hanta M212F	GTICCIGARAGYTCITGYAACATGG
	Muridae_hanta M483F	TICAIKCITGYAAYATGATGAAAAG
	Muridae_hanta M510R	CAICTTTTCATCATITTRCAIGMRTG
	Muridae_hanta M609R	TTITCIGGIACAAARCAYTTYCCTTC

	Muridae_hanta M903R	TCTTCICCAGIIARRTCATGRTCTTC
	Muridae_hanta M1199F	GCATCITGTGARGCITTYTCIGARGG
	Muridae_hanta M1652F	AARACWAARGGITCIATGGTITGTG
	Muridae_hanta M1754F	TACTGYTTTACICAYTGTGARCCIAC
	Muridae_hanta M1833F	GGGAIGATTTAAAIAARACWRTIAC
	Muridae_hanta M2661R	TCACCTGGITCWCCAAAITGRCAKG
	Muridae_hanta M2742R	GTTGTIGCAAAITTICAYTTYTTYC
	Muridae_hanta M3090R	GCACCITARCAIATIGCCWTRTCAC
	Muridae_hanta M3294R	CCACAYTSIGGIGCMCCATCATCATAIAC
	THAIV-like M2003F	CCACTCTGGACWGAYAATGCWCATGG
	THAIV-like M2030F	ATTGGITCWGTYCCIATGCAYACTG
	THAIV-like M2301F	TCAAAGTGRCACTTGGCTGTATGCCAAGG
	THAIV-like M2331F	GCCCAACTATTYTCATAYTCRTAATC

Table S3. Lists of primers used for the amplification and sequencing of L segment of Lanka virus, SA108, and Thailand virus strain Thai-749.

	Primer name	Sequence (5'-3')
Lanka virus specific primers	Lanka L260R	TCTAAAGCAGTAATTACATTATTAGG
	Lanka L299R	AAGGTTTTCCCACTCGGATGATCTGG
	Lanka L1956R	AAACCCAGAATATAGTGATGTAACAGCAGG
	Lanka L2033R	CTTTTAATGTTATAGTAAATGAATACTTC
	Lanka L2981F	CCGCAAAATTTAGAAGATTTACAGC
	Lanka L3013F	CACAATGGGCTGTCTGATAACAAGC
	Lanka L3271R	TAGCTTTGAAGAGAAGTGACATACC
	Lanka L3313R	ATTCAAAAAAGCAATCAAGCTCAGG
	Lanka L4303F	TGGGTCACATTTAGAGAGGTGCTTGCAGC
	Lanka L4333F	GCAAACAGCTTTGCAGAGAACTATGAACC
	Lanka L4927F	TTGTGTATTGAAGTCTGGAGATGGGC
	Lanka L4978F	GAATGGTTCCATGCGTTGTGGTTTG
Muridae-borne and THAIV-like primers	Muridae_hanta L1F	TAGTAGTAGACICCIDAARIRACAA
	Muridae_hanta L37F	GATGGAKAAATAYGGAAATWCAC
	Muridae_hanta L149F	ATTGTTGACCARATGATWAARCATG
	Muridae_hanta L642F	GAAGARAGAGCTGCAYTAGAAGCMATG
	Muridae_hanta L670R	AACATKGCTTCTARTGCAGCTCTYTC
	Muridae_hanta L1440F	AAGAAGACMACWGCATGGCAYATMGC
	Muridae_hanta L1501R	GCAATCAARCTYTCWGTWATATCCC
	Muridae_hanta L1876F	TTTGYCAAAAGATGAAAYTVTGTGC
	Muridae_hanta L2134R	CCRCTTGCACCAACWGTWGATTGGTC

	Muridae_hanta L3556F	TCWACATTTTTTGARGGTTGTGCTG
	Muridae_hanta L3864R	TCCATTATTGACATWGCACCAYTYCC
	Muridae_hanta L4218F	TAACAGCWATGACHATGCARTCACC
	Muridae_hanta L4434R	TTCCATGCATAYTCTTTWGARAATG
	Muridae_hanta L4577F	GTAACAGTWGAKGAAATGTCWGATG
	Muridae_hanta L5091R	AATGCTGCACAYTGRATYTCTGGATC
	Muridae_hanta L5153F	TATTCAGGKAARCARTATGATGCATATTG
	Muridae_hanta L5184R	ACACAATATGCATCATAYTGYTTMCC
	Muridae_hanta L5192F	TATAATGAAGWRACAAAGCTWTATG
	Muridae_hanta L5489R	CACAGTTYTTRTAWACAATACATGG
	Muridae_hanta L5650F	AGTTGATGCWGTRAGTAATGTRTGG
	Muridae_hanta L6033R	GCTTCTAYATCWATATTYTCAAAATC
	Muridae_hanta L6535R	TAGTAGTAGRCTCCGSAAAATGAAAAWG
	THAIV L301R	TTAGGGTCTTCCCACTAGGATGGTC
	THAIV L258R	TAACGCAGTTATCACATTGTTTGGG
	THAIV L6338F	AGTTTGATGCAATTGACAGGGAAGC
	THAIV L6386F	GAATCATTCCTGAGGATGTTATTCC

Table S4. Details of the accession numbers of *cytb* sequences and hantavirus genome sequences obtained from *Mus booduga* and *Rattus rattus* animals in this study.

Sample ID	Species	*cytb* sequences	Hantavirus sequences		
			S	M	L
PR98	*M. booduga*	LC556235	LC553716	LC553717	LC553718
PR99	*M. booduga*	LC556236	-	-	-
PR105	*M. booduga*	LC556237	-	-	-
PR106	*M. booduga*	LC556238	LC553719	LC553720	LC553721
PR108	*R. rattus*	LC556239	LC553722	LC553723	LC553724
PR110	*M. booduga*	LC556240	LC553725	LC553726	LC553727
PR111	*M. booduga*	LC556241	-	-	-
PR112	*M. booduga*	LC556242	-	-	-
PR113	*M. booduga*	LC556243	LC553728	LC553729	LC553730
PR114	*M. booduga*	LC556244	-	-	-
PR115	*M. booduga*	LC556245	-	-	-
PR116	*M. booduga*	LC556246	LC553731	LC553732	LC553733

Table S5. Nucleotide and amino acid identities of S segment ORF and nucleocapsid protein of novel viruses with representative Muridae-borne hantaviruses.

	Nucleotide %							
	THAIV	ANJZV	SA108	Lanka	Seoul	Hantaan	Dobrava	Puumala
THAIV	-	82.3	80.3	78.1	77.2	74.8	73.9	63.8
ANJZV	96.7	-	86.3	79.4	75.1	75.1	74.0	64.2
SA108	96.7	98.4	-	80.1	75.3	74.4	74.0	62.5
Lanka	92.1	93.2	93.0	-	75.0	74.0	72.6	62.6
Seoul	86.7	86.2	86.5	85.8	-	74.6	74.5	62.0
Hantaan	83.7	84.4	84.4	84.1	83.2	-	74.0	62.8
Dobrava	82.8	83.2	83.2	83.2	81.6	83.0	-	63.4
Puumala	62.4	62.4	62.4	61.4	61.9	60.5	61.2	-
	Amino acid %							

Thailand virus strain Thai-749 (AB186420), Anjozorobe virus strain- Anjozorobe/Em/MDG/2009/ATD49 (KC490918), Hantaan virus strain-HTN76-118 (M14626), Seoul virus strain-80-39 (AY273791), Dobrava virus strain-Dobrava-Belgrade (L41916), Puumala virus strain-Sotkamo (X61035). S segment sequences of Lanka virus (LC553716), and SA108 (LC553722) were used for the comparison.

Table S6. Nucleotide and amino acid identities of M segment ORF and glycoprotein precursor of novel viruses with representative Muridae-borne hantaviruses.

	Nucleotide %							
	THAIV	ANJZV	SA108	Lanka	Seoul	Hantaan	Dobrava	Puumala
THAIV	-	79.6	79.9	76.6	74.5	71.7	71.6	59.7
ANJZV	91.4	-	87.1	76.9	74.2	71.3	71.5	59.4
SA108	91.9	96.9	-	76.9	73.9	71.2	71.2	59.4
Lanka	85.4	86.7	87.2	-	72.3	71.2	70.7	59.4
Seoul	82.0	82.9	83.3	79.7	-	72.0	71.2	60.7
Hantaan	77.1	77.5	78.3	76.8	77.1	-	71.4	60.1
Dobrava	76.7	77.2	78.2	75.7	77.2	77.4	-	60.5
Puumala	53.8	53.3	53.5	53.6	53.8	53.8	53.3	-
	Amino acid %							

Thailand virus strain Thai-749 (L08756), Anjozorobe virus strain- Anjozorobe/Em/MDG/2009/ ATD49 (KC490919), Hantaan virus strain-HTN76-118 (M14627), Seoul virus strain-80-39 (S47716), Dobrava virus strain-Dobrava-Belgrade (L33685), Puumala virus strain-Sotkamo (X61034). M segment sequences of Lanka virus (LC553717) and PR108 (LC553723) were used for the comparison.

Table S7. Nucleotide and amino acid identities of L segment ORF and L-protein of novel viruses with representative Muridae-borne hantaviruses.

	Nucleotide %							
	THAIV	ANJZV	SA108	Lanka	Seoul	Hantaan	Dobrava	Puumala
THAIV	-	79.3	79.5	79.2	76.3	74.2	74.9	67.0
ANJZV	94.9	-	86.5	79.5	76.2	74.8	74.8	66.6
PR108	95.0	97.8	-	79.7	76.5	74.7	75.4	67.2
Lanka	94.0	94.3	94.5	-	76.7	74.0	74.2	66.9

Seoul	89.1	88.8	88.7	88.3	-	74.5	75.0	67.1
Hantaan	85.1	84.7	84.9	84.8	85.1	-	74.7	67.0
Dobrava	85.9	86.0	86.1	85.6	85.6	85.1	-	66.9
Puumala	68.0	68.4	68.5	68.5	68.6	69.0	69.4	-
Amino acid %								

Thailand virus strain Thai-749 (LC553715), Anjozorobe virus strain- Anjozorobe/Em/MDG/2009/ ATD49 (KC490922), Hantaan virus strain-HTN76-118 (X55901), Seoul virus strain-80-39 (X56492), Dobrava virus strain-Dobrava-Belgrade (JQ026206), Puumala virus strain-Sotkamo (Z66548). L segment sequences of Lanka virus (LC553718), and PR108 (LC553724) were used for the comparison.

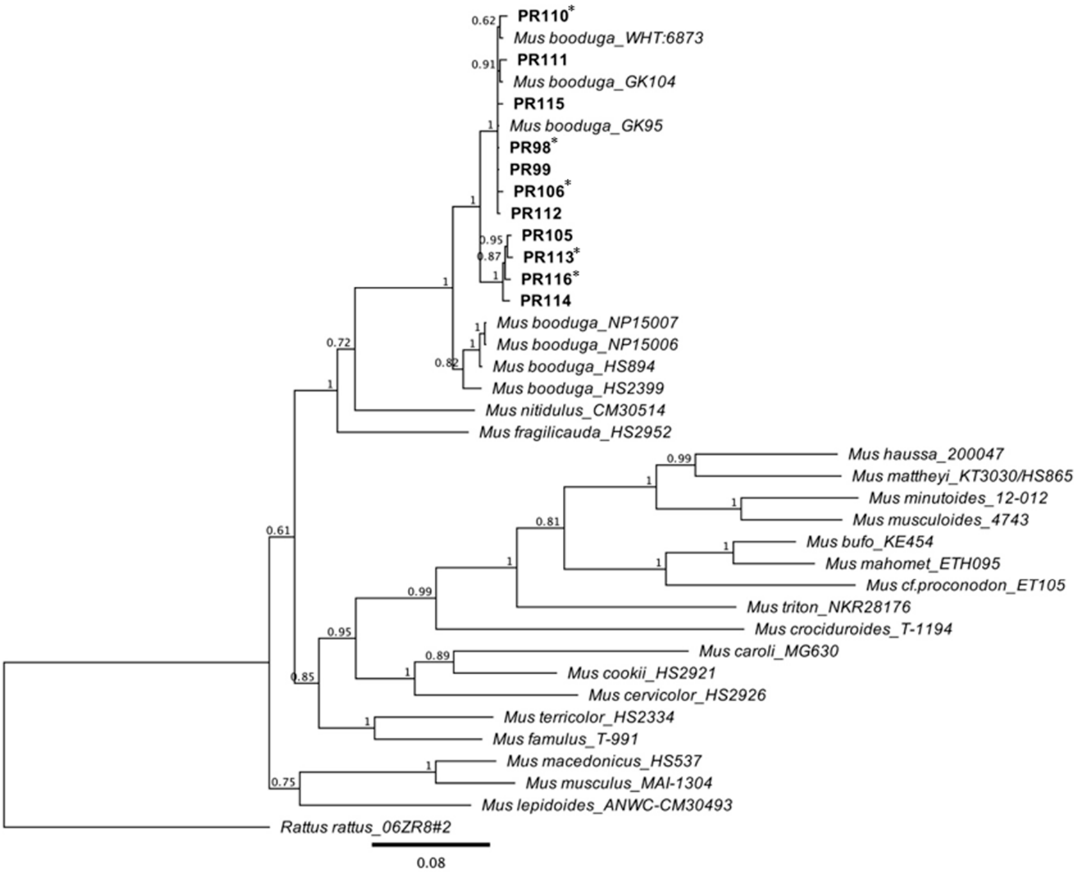

Figure S1. Phylogenetic tree based on *cytb* sequences of *Mus booduga* samples obtained in this study (in boldface) and other *Mus* spp. retrieved from databases.

The scale bar indicates a sequence divergence of 0.08. Numbers above nodes indicate the Bayesian posterior probability values. *M. booduga from Sri Lanka*: GK104 (LC426363), GK95 (LC426362), WHT-6873 (KY697998), *M. booduga from Nepal:* NP15007 (KY587424), NP15006 (KY587423), HS894 (AB125761), *M. booduga from India:* HS2399 (AB125760). *M. nitidulus*: CM30514 (AB269818), *M. fragilicauda*: HS2952 (AB125780), *M. haussa*: 200047 (AJ875071), *M. mattheyi*: KT3030/HS865 (AB125781), *M. minutoides*: 12-012 (LM994813), *M. musculoides*: 4743 (AJ875075), *M. bufo*: KE454 (KJ935783), *M. mahomet*: ETH095 (KJ935795), *M. cf. proconodan*: ET105 (KJ935769), *M. triton*: NKR28176 (KJ935746), *M. crociduroides*: T-1194 (AJ698878), *M. caroli*: MG630 (AB109795), *M. cookii*: HS2921 (AB125767), *M. cervicolor*: HS2926 (AB125764), *M. terricolor*: HS2334 (AB125776), *M. famulus*: T-991 (AJ698872), *M. macedonicus*: HS537 (AB125770), *M. musculus*: MAI-1304 (LC325143), *M. lepidoides*: ANWC-CM30493 (AB262414), and *Rattus rattus*: 06ZR8#2 (AB752981).

*Virus positive rodent samples

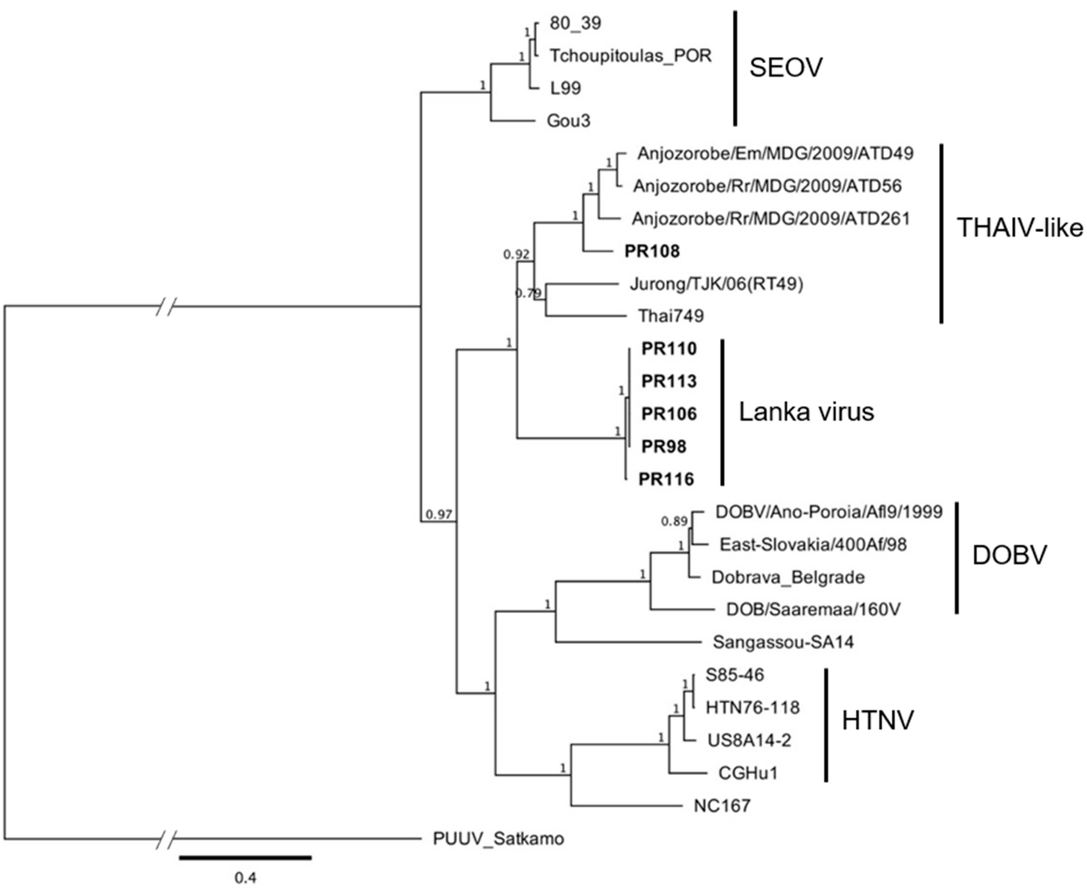

Figure S2. Phylogenetic tree based on M segment ORF sequences representing Muridae-borne hantaviruses and new-found viruses (in boldface) in this study.

The scale bar indicates a sequence divergence of 0.4. Numbers above nodes indicate the Bayesian posterior probability values. Hantaan: S85-46 (AF288658), HTN76-118 (M14627), US8A14-2 (KU207204), CGHu1 (EU092222), Dabieshan: NC167 (AB027115); Seoul: 80-39 (S47716), Tchoupitoulas-POR (KU204959), L99 (AF035833), Gou3 (AF145977); Dobrava: DOBV/Ano-Poroia/Afl9/1999 (AJ410616) Dobrava-Belgrade (L33685), DOB/Saaremaa/160V (AJ009774). Sangassou: SA14 (JQ082301); THAIV: Thai-749 (L08756), ANJZV-strain Anjozorobe/Em/MDG/2009/ ATD49 (KC490919), ANJZV-strain Anjozorobe/Rr/MDG/2009/ ATD56 (KC490921), ANJZV-strain Anjozorobe/Rr/MDG/2009/ATD261 (KC490920), Jurong-strain TJK/06/RT49 (GQ274938); Puumala: Sotkamo (X61034).

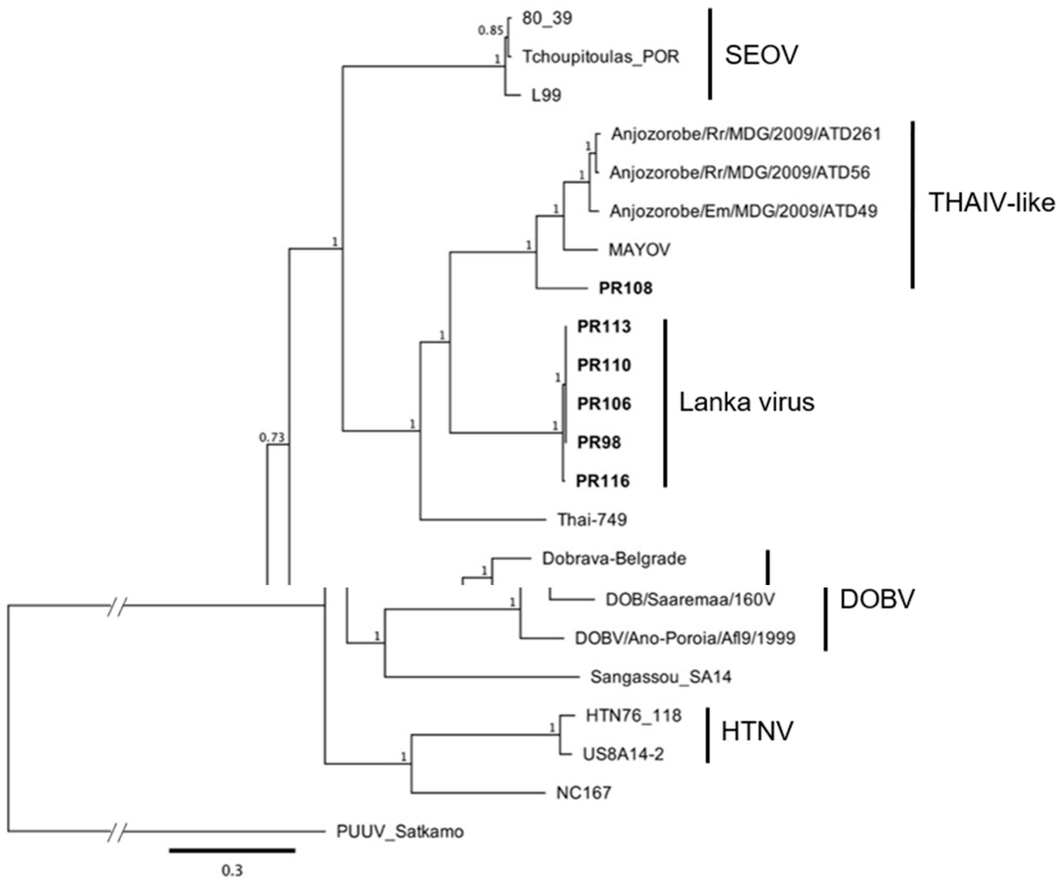

Figure S3. Phylogenetic tree based on L segment ORF sequences representing Muridae-borne han-taviruses and newfound viruses (in boldface) in this study. The scale bar indicates a sequence di-vergence of 0.3. Numbers above nodes indicate the Bayesian posterior probability values. Hantaan: HTN76-118 (X55901); Dabieshan: NC167 (DQ989237); Seoul: 80-39 (X56492), Tchoupitoulas-POR (KU204958), L99 (AF288297); Dobrava: DOBV/Ano-Poroia/Afl9/1999 (AJ410617) Dobrava-Belgrade (JQ026206), DOB/Saaremaa/160V (AJ410618). Sangassou: SA14 (JQ082302); THAIV: Thai-749 (LC553715), ANJZV-strain Anjozorobe/Em/MDG/2009/ ATD49 (KC490922), ANJZV-strain Anjozorobe/Rr/MDG/2009/ ATD56 (KC490923), ANJZV-strain Anjozorobe/Rr/MDG/ 2009/ ATD261 (KC490924), Mayotte-strain MAYOV (KU587796); Puumala: Sotkamo (Z66548).
